# Impact of Simulation on the Development of Nursing Students' Competence in Adult Cardiopulmonary Resuscitation

**DOI:** 10.7759/cureus.72722

**Published:** 2024-10-30

**Authors:** Ghizlane El Ougli, Brahim Boukatta, Abderrahim El Bouazzaoui, Soumaya Touzani, Nawfal Houari, Samira El Fakir, Nabil Kanjaa

**Affiliations:** 1 Faculty of Science and Technology, Sidi Mohamed Ben Abdellah University, Fez, MAR; 2 Laboratory of Anatomy, Surgery and Anesthesiology, Faculty of Medicine, Pharmacy and Dental Medicine of Fez, Sidi Mohamed Ben Abdellah University, Fez, MAR; 3 Department of Medical Education, Higher Institute of Nursing Professions and Health Technology, Fez, MAR; 4 Department of Anesthesiology and Intensive Care, Faculty of Medicine, Pharmacy and Dental Medicine of Fez, Sidi Mohamed Ben Abdellah University, Fez, MAR; 5 Department of Epidemiology and Public Health, Faculty of Medicine, Pharmacy and Dental Medicine of Fez, Sidi Mohamed Ben Abdellah University, Fez, MAR

**Keywords:** adult cardiac arrest, cardiopulmonary resuscitation, knowledge, non-technical skills, nursing students, simulation, technical skills

## Abstract

Background: Cardiopulmonary resuscitation (CPR) training is crucial for nursing students as future nurses are the first responders in the ambulatory and hospital care chain. Simulation-based learning has been shown to be more effective in acquiring the knowledge and technical and non-technical skills required for CPR. The present study aims to evaluate the impact of high-fidelity simulation-based adult CPR training on the acquisition and retention of knowledge and the development of technical and non-technical skills of nursing students at the Higher Institute of Nursing Professions and Health Techniques in Fez, Morocco.

Methods: We conducted an interventional study with 49 nursing students. Twenty-five students (51.02%) in the simulation group received both traditional CPR training (theoretical lecture and demonstration of procedures) and simulation CPR training, while 24 students (48.97%) in the control group received only traditional training. Data were obtained using a questionnaire on theoretical knowledge of CPR, a technical skills assessment grid, and the Team Emergency Assessment Measure (TEAM) for non-technical skills.

Results: The post-test scores for theoretical knowledge immediately after simulation were significantly higher for the simulation group (16.41±1.73 out of 20) than for the control group (13.15±1.96 out of 20). Mean CPR knowledge retention scores 30 days after training were significantly higher in the simulation group (15.60±1.71) compared with the control group (11.38±1.93). Assessment of technical skills on a high-fidelity mannequin showed a considerable advantage for the simulation group (30.88±1.64 out of 34) over the control group (18.00±0.78 out of 34). In addition, the results demonstrated a significant difference between the TEAM score of the simulation group (37.84±2.67 out of 44) and the control group (22.33±0.96 out of 44) and a significant difference between the Global Team Performance of the simulation group (8.16±0.68) out of 10 and the control group (4.83±0.38).

Conclusion: The findings demonstrated that adult CPR training using high-fidelity simulation was superior to the traditional method with regard to knowledge acquisition and retention and the development of technical and non-technical skills in undergraduate nursing students.

## Introduction

In-hospital cardiac arrest (IHCA) represents a significant burden on healthcare systems worldwide [1]. Survival from sudden cardiac arrest rates vary between studies and have steadily increased over the last decade [2]. It depends on precise resuscitation and timely execution of vital procedures, such as early cardiac rhythm detection, prompt defibrillation, efficient basic life support, and post-resuscitation care [3]. However, inadequate team responses resulting in delayed CPR may be fatal. In fact, every minute of delay in CPR equals a 7-10% decrease in survival chances [4].

Nurses play an essential role in effective IHCA management as first responders, being often the first to detect cardiac arrest and initiate cardiopulmonary resuscitation (CPR) [5].

Indeed, CPR is a highly skilled procedure, and the proficiency of healthcare professionals has a major role in its outcome [6]. A study that looked at residents' skills and motivation in subjects relevant to CPR, such as nursing, underscored the need of CPR training for medical professionals [7]. It is evident that suitable and successful training can aid them in completing the life-saving procedure [8]. A few hours of theoretical and practical CPR instruction can prevent tragic deaths [7].

Additionally, as prompt diagnosis and treatment of cardiac arrest doubles victim survival rates, nursing students should be proficient in the rapid response necessary for treating cardiac arrest [9]. In parallel, previous studies have found associations between advanced CPR and an increase in the rate of return of spontaneous circulation [10].

In healthcare environments, poor-quality CPR constitutes avoidable harm [11]. Nevertheless, there is evidence that students' and nurses' performance regularly falls short of international criteria, indicating serious quality deficiencies [12].

Liou et al. also observed that nursing students often face challenges and obstacles in comprehending the course content and developing the clinical skills related to CPR [13]. Some research attributes nursing students' deficient skills and poor performance in the precise conduct of CPR to inadequate training and nursing students' dissatisfaction with teaching methodology [14,15]. In addition, a qualitative survey conducted by Dziurka et al. found that a significant proportion of nursing students reported discontent with the teacher-centered aspect of the pedagogical approach, which focuses solely on theoretical knowledge [16]. According to a number of research, nursing graduates' and students' knowledge and performance tend to deteriorate over time [17]. In order to guarantee patient safety and enhance learners' confidence in executing clinical interventions such as CPR, professors must deliver precise instruction in clinical competencies using innovative training techniques [18].

The adoption of effective and appropriate teaching strategies has received scant attention, despite the critical relevance of CPR instruction. Consequently, the current urgency emphasizes the critical need to raise the standard of CPR training rather than just increasing its availability [19].

Because students will eventually work as healthcare professionals and may encounter cardiac arrest, it is imperative that effective training measures be implemented to overcome the constraints placed on nurses [11].

A long-standing issue is the acquisition and retention of CPR skills and knowledge [20]. A traditional course or training program may be able to provide satisfactory theoretical levels; nevertheless, research indicates that knowledge and skills tend to be forgotten or lose value with time [21].

In order to enhance healthcare practitioners' skills and improve knowledge retention, innovative teaching methods, such as simulation, have gained popularity recently in training in CPR [22]. High-fidelity simulations used in standardized simulation-based educational procedures have been shown to support active learning in a repeatable and secure setting and to enhance teamwork, resuscitation knowledge, and skills [23]. High-fidelity simulator use in nursing education has been shown in multiple studies to positively impact knowledge acquisition as well as the development of technical and non-technical skills necessary for safe and efficient crisis management that benefits patients [24]. These non-technical skills include cognitive and social skills that complement and enhance clinicians' technical skills, such as decision-making, planning, communication, teamwork, and leadership [25,26].

Compared to traditional teaching, high-fidelity simulation appears to be a powerful educational tool that promotes better knowledge retention over time [27]. Thus, the effectiveness of CPR training can be improved by increasing the frequency of high-fidelity simulation training, which can protect against the deterioration of knowledge and skills [28]. This is crucial as the retention of CPR knowledge and skills is widely recognized as an important factor in real-life resuscitation [29].

In our context, few research have been conducted on the integration of high-fidelity simulation into an emergency and critical care program in nursing education, with the aim of fostering knowledge acquisition and retention and the development of technical and non-technical skills in nursing students. On this basis, this study aims to evaluate the effect of a high-fidelity simulation intervention on the acquisition and retention of knowledge and the development of technical and non-technical skills of nursing students in recognizing and responding to a critical nursing situation example of cardiorespiratory arrest in adults.

## Materials and methods

Study population

The study included all nursing students enrolled in the fourth semester of the professional license cycle (from the options: Nurse in Emergency and Intensive Care (NEIC) and Nurse in Anesthesia and Reanimation (NAR) at the Higher Institute of Nursing Professions and Health Techniques (HINPHT) of Fez, Morocco). The adult CPR course is part of the resuscitation module. The students who had not yet received the adult cardiopulmonary arrest course were included, whereas students who had already received the adult CPR course or practiced it as part of a clinical or simulation practice were excluded.

Out of the 58 eligible students (meeting these criteria), 49 students were available and included in this study. 

Study design and setting

This study was designed as an interventional study with an equivalent control group, before and after simulation training, to compare differences in knowledge acquisition and retention and technical and non-technical skill development in adult CPR between the simulation and control groups. The simulation group was trained using high-fidelity simulation, while the control group received only traditional theoretical training in performing in-hospital adult CPR. The training lasted three days and was carried out in a single location: the simulation laboratory at HINPHT, Fez. The teaching team consisted of a senior lecturer in anesthesia and intensive care at the Faculty of Medicine, Pharmacy and Dentistry of Fez and the principal investigator (GE).

**Figure 1:**
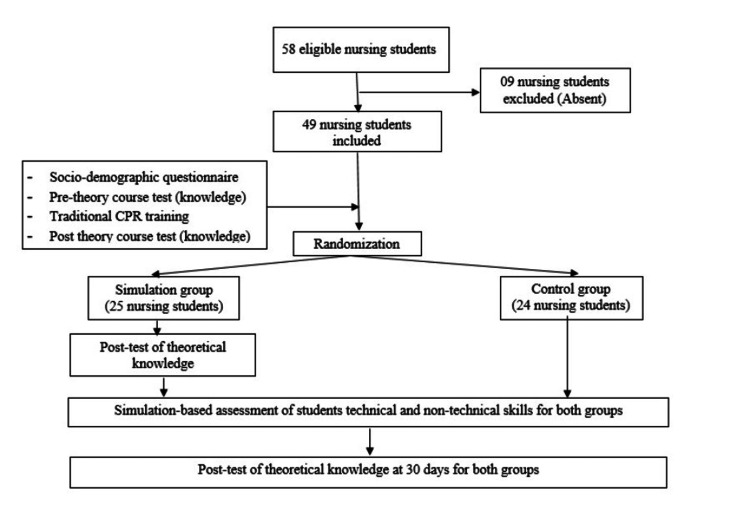
Sequence of events of the study CPR: cardiopulmonary resuscitation

The study was organized into seven steps (see Figure 1).

The first step consisted of administering the student socio-demographic questionnaire to all participants (with an additional question verifying their prior knowledge and practical experience of adult in-hospital CPR) and administering the adult in-hospital CPR knowledge questionnaire (pre-test). The questionnaires were completed under the same code that each student chose during the training course.

In the second step, theory and practical training (traditional training) was provided for the group of 49 students. This training covered all the parts of the survival chain of adult in-hospital CPR (recognition, alert, ventilation, external cardiac compressions, defibrillation, etc.). The session took place over one day. The aim of this stage was to homogenize the students' knowledge.

The third step was devoted to assessing students' acquisition of theory (post-test immediately after the theory course). This assessment was carried out for all students (49 students) using the same questionnaire and code used previously.

In the fourth step, randomization was carried out by drawing lots within the group of students, dividing them into two groups. Each student drew a number at random: if he drew the number 1, he was automatically placed in group 1, the group that benefited from the simulation (simulation group, n=25; 51.02%), and if he drew the number 2, he was placed directly in group 2, the group that did not benefit from the simulation (control group, n=24; 48.97%). Subsequently, each group of students was split into subgroups of four to five students, ending up with 12 subgroups: six simulation subgroups (five subgroups of four students and one subgroup of five students) and six control subgroups of four students in each subgroup. Thus, both the simulation and control groups had the same number of students for each option (the emergency and intensive care nursing option and the anesthesia and resuscitation nursing option). At the end of this stage, the students were divided into two comparable groups (simulation group and control group) in terms of the number of participants, level of knowledge (based on the immediate post-test score), practical experience in CPR, and level of study (fourth semester). The composition and running order of the teams was decided randomly, and a number was assigned to each subgroup. 

The fifth step was the simulation run for the simulation group on a scenario involving a high-fidelity mannequin simulator. The scenario chosen was that of a patient, aged 67, who presented to the emergency department with chest pain, slowly progressive shortness of breath without malaise, or loss of consciousness. For the first few minutes of the simulation, the simulated patient was conscious, and the students were able to communicate with him, with the patient's voice being provided by the trainer. Beyond that, the patient lost consciousness due to the onset of cardiopulmonary arrest. The scenario provided for recovery from cardiorespiratory arrest after the third defibrillation.

All participants were active learners, to avoid observer bias in the measurement of self-evaluation results. The simulation session (briefing-situation-debriefing) was carried out for each subgroup. The scenario included a nurse facilitator (provided by the principal investigator), who helped move the scenario forward when necessary. At the end of this session, all students in the simulation group (n=25; 51.02%) completed the theory knowledge questionnaire (post-test: immediately after the simulation), using the same code they had been using since the start of the training.

The sixth step consisted in a simulation-based assessment of students' technical and non-technical skills in both randomized groups, using a high-fidelity mannequin. The scenario chosen was the same one used in the previous stage. During this stage, each of the subgroups in the simulation and control groups was assessed using the technical skills assessment chart and the Team Emergency Assessment Measure (TEAM) non-technical skills grill.

In the last step, a retention test of theoretical CPR knowledge was carried out in the seventh stage at 30 days post-training for both simulation and control groups using the same questionnaire for theoretical knowledge in CPR and the same code used by the students since the start of the training course.

Data collection

Four data collection tools were used in this study: The first is the student socio-demographic data questionnaire, which included characteristics of students' sex, age, nationality, option, prior CPR knowledge, and practical CPR experience in clinical practice or a simulation session.

The second is the CPR knowledge questionnaire (pre-test and post-test) prepared by the principal investigator on the basis of the American Heart Association (AHA) [30,31] and European Resuscitation Council (ERC) [32] guidelines and with the advice of professors in higher education at the Faculty of Medicine, Pharmacy and Dental Medicine specializing in anesthesia and intensive care and experts in CPR. The questionnaire consisted of 20 multiple-choice questions (1 point was awarded for the correct answer and 0 for the wrong answer). The questionnaire was marked with a grade of 20/20 and completed under the same code name chosen by each student during training, to enable comparison of results.

The third is the technical skills evaluation chart focusing on technical skills in adult in-hospital CPR. It was developed by local experts on the basis of current literature and international recommendations [30-32]. The chart includes 34 items covering the different aspects of CPR: diagnosis, alert, basic CPR, and defibrillation. The technical skills evaluation chart included three additional items concerning the preparation and administration of medication, compliance with asepsis and hygiene rules, and compliance with work attire. The following scoring method was adopted: Each group received 1 point for each of the 34 skill elements that they performed correctly and in the right order, 0.5 for performing it incompletely and needing to improve, and no points for not performing the skill element or performing it incorrectly. CPR technical skill scores ranged from 0 to 34 correct elements of cardiopulmonary arrest management on a rhythm that was initially non-shockable and then shockable.

To ensure the methodological robustness of our study, we developed the CPR knowledge questionnaire and the CPR technical skills assessment grid in alignment with guidelines established by the AHA [30,31] and the ERC [32]. These tools comply with AHA and ERC recommendations, ensuring that the data collected is relevant and standardized according to international best practice. We also took into account local expertise in the field, consulting experts and adapting the tools to suit the specific context of our study. This ensures that the tools are culturally and contextually appropriate, increasing the relevance and applicability of the results.

Lastly, the TEAM scale (validated French version) was used to assess non-technical skills. This is a validated descriptive global scale for assessing the performance of an interprofessional team in a critical care setting [33-36]. It consists of 11 items, divided into three categories: leadership (sum of items 1 and 2), teamwork (sum of items 3-9), and task management (sum of items 10 and 11). Each element describes a variety of possible behaviors, rated from 0 (never/almost never) to 4 (always/almost always), and by combining the 11 elements, an overall TEAM score from 0 to 44 is obtained. The TEAM scale also includes a Global Team Performance (GTP) score, which is different from the overall TEAM score and is rated on a scale of 1-10. This GTP score enables a global assessment that goes beyond technical skills and critical situation management [36]. The researcher obtained written consent from the author of the TEAM instrument (French version) for its use in this study.

Data analysis

Statistical data were analyzed using IBM SPSS Statistics for Windows, V. 26.0 (IBM Corp., Armonk, NY, USA). Quantitative data were expressed as means and standard deviations, while qualitative data were expressed as counts and percentages.

To examine changes in theoretical knowledge between different study times in the simulation and control groups, we used the t-test for paired series. This test is particularly suitable for comparing two measurements taken on the same subjects, as it allows us to determine whether the means of the two sets of data are significantly different from each other.

The t-test for independent series was used to compare mean scores for theoretical knowledge and technical and non-technical skills between simulation and control groups. This test is suitable for comparing the means of two independent groups, checking whether the differences observed are statistically significant, and p<0.05 was considered significant.

The use of these t-tests is justified by their ability to assess significant differences in group means while accounting for intra-group and inter-group variation, which is essential for the validity of our conclusions.

Ethical considerations

This study is approved by the Hospital-University Ethics Committee of Fez, Laboratory of Anatomy, Surgery and Anesthesiology, Faculty of Medicine, Pharmacy and Dental Medicine of Fez, Sidi Mohamed Ben Abdellah University, Fez, Morocco, on December 15, 2022 (N°32/22), and the Regional Directorate of Health and Social Protection of Fez Meknes, Morocco, on October 26, 2022 (authorization N°5997). Prior to data collection, all participating students provided written informed consent. Responses were kept strictly confidential and participation was entirely voluntary. Every participant was free to leave the research at any moment.

## Results

Forty-nine nursing students were included in the study and randomly assigned to either the simulation group (n=25 (51.02%): 14 (56%) NAR and 11 (44%) NEIC) or the control group (n=24 (48.97%): 14 (58.33%) NAR and 10 NEIC (41.66%)). The majority of responders were female (n=37; 75.5%) and the mean age was 19.92±0.99 years old. The majority of participants were Moroccan and three (6.1%) students were foreigners. All study participants reported that they had no previous knowledge or practical experience of adult in-hospital CPR (Table 1).

**Table 1:** Socio-demographic characteristics of participants n: sample size; M: mean; SD: standard deviation; NAR: Nurse in Anesthesia and Reanimation; NEIC: Nurse in Emergency and Intensive Care

Variables		Total (%) (n=49)
Sex	Female	37 (75.5%)
	Male	12 (24.5%)
Nationality	Moroccan	46 (93.9%)
	Foreign	3 (6.1%)
Option	NAR	28 (57.14%)
	NEIC	21 (42.85%)
Age (M±SD)		19.92±0.99

In the simulation group, there was a significant improvement in the average scores for theoretical knowledge overall. However, there was a significant decrease in the mean scores for theoretical knowledge between the post-test conducted immediately after the simulation training and the post-test administered 30 days later (Table 2).

**Table 2:** Evolution of theoretical knowledge in the simulation group M: mean; SD: standard deviation

Group	Pre-theory course test (score out of 20) (M±SD)	Immediate post-theory course test (score out of 20) (M±SD)	Immediate post-simulation test (score out of 20) (M±SD)	Post-simulation test at 30 days (score out of 20) (M±SD)	P-value
Simulation	7.02±2.11	13.72±2.21			<0.001
	7.02±2.11		16.41±1.73		<0.001
	7.02±2.11			15.60±1.71	<0.001
		13.72±2.21	16.41±1.73		<0.001
		13.72±2.21		15.60±1.71	<0.001
			16.41±1.73	15.60±1.71	<0.001

In the control group, there was a significant improvement in the mean scores at the immediate post-test and the post-test 30 days later compared with the mean scores obtained at the pre-test. However, there was a significant decrease in mean knowledge scores between the post-test immediately after the theory course and the post-test 30 days later (Table 3).

**Table 3:** Evolution of theoretical knowledge in the control group M: mean; SD: standard deviation

Group	Pre-theory course test (score out of 20) (M±SD)	Immediate post-theory course test (score out of 20) (M±SD)	Post-theory course test at 30 days (score out of 20) (M±SD)	P-value
Control	7.13±1.87	13.15±1.96		<0.001
	7.13±1.87		11.38±1.93	<0.001
		13.15±1.96	11.38±1.93	<0.001

The results of the pre-test for theoretical knowledge of CPR indicated no statistically significant difference in mean scores between the simulation and control groups (p=0.84). Furthermore, the post-test results for theoretical knowledge of CPR showed no statistically significant difference in mean scores between the simulation and control groups (p=0.35).

When comparing CPR knowledge acquisition scores, students in the simulation group, who received both the theoretical CPR course and the simulated cardiopulmonary arrest experience, had significantly higher scores (p<0.001), as measured by the second post-test of theoretical CPR knowledge, than students in the control group, who received only the theoretical CPR course.

During the retention phase of the study, 30 days later, the students were asked to retake the same multiple-choice test to measure the retention of theoretical CPR knowledge. The mean CPR knowledge score for the 25 participants in the simulation group was 15.60±1.71. The mean CPR knowledge score for the 24 control group participants was 11.38±1.93. The mean CPR knowledge retention scores 30 days later after training were significantly higher (p<0.001) for the experimental group than for the control group (Table 4).

**Table 4:** Comparison of theoretical CPR knowledge scores between simulation and control groups (before and after) CPR: cardiopulmonary resuscitation; M: mean; SD: standard deviation

Simulation group (score out of 20) (M±SD)	Control group (score out of 20) (M±SD)	P-value
Pre-test score		
7.02±2.11	7.13±1.87	0.84
Post-test score (immediately after the theoretical course)		
13.72±2.21	13.15±1.96	0.35
Post-test score (immediately after the simulation)	Post-test score (immediately after the theoretical course)	<0.001
16.41±1.73	13.15±1.96	
Post-test score at 30 days		
15.60±1.71	11.38±1.93	<0.001

During the technical skills evaluation phase in CPR, the mean score of the simulation group was 30.88±1.64, and the mean score of the control group was 18.00±0.78. The students in the simulation group demonstrated a significant increase in technical skills in CPR (p<0.001) during the evaluation phase in the cardiac arrest scenario after simulation-based training (Table 5).

**Table 5:** Comparison of technical skills in CPR between the simulation group and the control group CPR: cardiopulmonary resuscitation; M: mean; SD: standard deviation

Technical skills scores		Simulation group (M±SD)	Control group (M±SD)	P-value
Diagnostic/6	Evaluate consciousness	5.84±0.37	3.33±0.76	<0.001
	Evaluate breathing: clearance of the upper airways			
	Evaluate breathing: see, listen, feel			
	Evaluate breathing: duration <10 seconds			
	Check carotid pulse			
	Check pulse: duration <10 seconds			
Alert the doctor early/1		0.76±0.25	0.25±0.25	<0.001
Start the timer/1		0.92±0.18	0.25±0.25	<0.001
External cardiac massage and ventilation/9	External cardiac massage: the first step of CPR=chest compressions	8.76±0.38	6.33±1.60	<0.001
	Patient's position: supine position			
	Patient's position: on a hard surface			
	Identify the appropriate location: lower half of the sternum			
	Positioning of the student			
	Apply a pressure of 100-120/min			
	Compress to a depth of 5-6 cm			
	Perform 30 compressions and two ventilations: 30/2			
	Ventilation: bag valve mask under O2 (15 L/min)			
Bring back the emergency cart/1		1.00±0.00	0.58±0.19	<0.001
Algorithm non-shockable rhythm/4	Recognize non-shockable rhythm	3.84±0.23	2.33±0.38	<0.001
	Administer adrenaline on medical prescription 1 mg intravenously			
	Administer adrenaline on medical prescription 1 mg/4 min			
	Reevaluate every two minutes			
Algorithm shockable rhythm/9	Recognize the shockable rhythm	6.76±1.31	3.08±1.39	<0.001
	Apply the semi-automatic defibrillator on medical advice 150-200 J			
	Apply the semi-automatic defibrillator on medical advice every two minutes			
	Administer adrenaline on medical prescription after the third shock			
	Administer adrenaline on medical prescription after the third shock: 1 mg intravenously			
	Administer adrenaline on medical prescription after the third shock: 1 mg/4 min			
	Administer amiodarone on medical prescription 300 mg after the third shock intravenously			
	Administer amiodarone on medical prescription 150 mg after the fifth shock intravenously			
	Reevaluate every two minutes			
Prepare and administer medications/1		1.00±0.00	0.58±0.19	<0.001
Respect aseptic and hygiene rules/1		1.00±0.00	0.66±0.24	<0.001
Respect work outfit/1		1.00±0.00	0.58±0.19	<0.001
Global score/34		30.88±1.64	18.00±0.78	<0.001

Statistical evaluations revealed that there was a significant difference (p<0.001) between the overall TEAM score of the simulation group and the control group (37.84±2.67 versus 22.33±0.96, respectively). This result showed that non-technical skills significantly improved following the simulation session. Teamwork, leadership, and task management were particularly significantly higher in the simulation group compared to the control group (Table 6).

**Table 6:** Comparison of non-technical skills between the simulation group and the control group M: mean; SD: standard deviation; TEAM: Team Emergency Assessment Measure; GPS: Global Performance Score

Categories of the TEAM scale	Simulation group (M±SD)	Control group (M±SD)	P-value
Overall TEAM score (0-44)	37.84±2.67	22.33±0.96	<0.001
Teamwork (0-28)	22.64±1.49	14.50±0.78	<0.001
Leadership (0-8)	7.68±0.74	3.83±0.38	<0.001
Task management (0-8)	7.52±0.77	3.83±0.38	<0.001
GPS (0-10)	8.16±0.68	4.83±0.38	<0.001

## Discussion

CPR necessitates ongoing concurrent instruction in theory and practice. Understanding CPR and performing it correctly are significantly correlated with the chances of surviving [37]. Therefore, CPR training based on simulation for future healthcare providers and first responders is crucial as it is considered as being more efficient. The results of our study revealed that the knowledge of the simulation group who received simulation training in adult CPR improved significantly compared to the control group (Table 4). According to a study by Aljohani et al., using simulation as a dynamic teaching technique aids in the development of students' knowledge regarding the application of clinical skills. Furthermore, simulation has a greater impact on enhancing nursing students' knowledge and confidence in executing critical care skills than both theoretical and practical training [38]. Moreover, simulation allows for the observation of students' deficiencies in skills and the identification and filling up of knowledge gaps in nursing theory [39]. Active learning through simulation encourages students to build on what they already know and increases their knowledge acquisition [40]. High-fidelity simulation can also help increase the confidence of nursing students to apply the skills in future clinical practice as simulation scenarios reflect reality [41].

Moreover, the results showed that the theoretical knowledge retention scores at 30 days were significantly higher in the simulation group (Table 4). This has been reported in previous studies using high-fidelity simulations compared to lower-fidelity approaches [42]. In addition, more concrete and active experiences like using simulation increase student knowledge retention and may even enhance future learning skills and patient outcomes [43].

Similar research has demonstrated that undergraduate nursing students can enhance their knowledge retention and proficiency in clinical judgment, decision-making, prioritization, communication, and teamwork using high-fidelity simulation [42].

Nevertheless, the results of this study showed a significant decrease in the average theoretical knowledge scores of both the simulation group (Table 2) and control group (Table 3) at 30 days. This may be explained by the lack of clinical practice and experience but also highlights the importance of continuous training both for students and by extent nurses (repeating simulation sessions, etc.). The literature shows that knowledge also increases with multiple simulation sessions [44]. Furthermore, research showing improved results with increased exposure to simulation-based learning makes it clear that simulation-based learning needs to be introduced early in the nursing program [45]. In fact, retention is improved when students are repeatedly exposed to the same material. In health professions training, such as nursing degrees, where students frequently fail to remember what they have learned, this strategy has proven to be particularly helpful in improving retention and comprehension of content [46]. 

It has also been discovered in the present study that the high-fidelity simulation approach is far more successful than the conventional training approach at raising the technical skill levels of nursing students (Table 5). The provision of a secure and regulated atmosphere that allows nursing students to fully engage in a clinical context is advantageous for the development and refining of their skills prior to engaging with patients [47]. Therefore, this may increase their confidence to apply their critical care skills more in a real clinical situation [48]. It has been demonstrated that through experience learning, reflection, and debriefing, high-fidelity simulation has been demonstrated to link theoretical nursing knowledge with practical clinical skills [49]. Consequently, simulation has been included in nursing curricula to better prepare students for their clinical experiences [50].

Although training for specific tasks guarantees the acquisition of skills and intra-individual performance, it does not address the problem of errors in communication or in the management of material or human resources within a team [51]. The majority of complications and accidents in healthcare are not due to a lack of knowledge or an inadequate technical procedure carried out by a caregiver but, more often than not, to non-technical skills that are often described as human factors [52], which include cognitive, social, and personal resources complementary to procedural know-how contributing to efficient and safe performance [53]. Indeed, in cases where a patient presents a critical clinical situation (known as a crisis situation), such as cardiopulmonary arrest, a group of specific non-technical skills are required. Known as "Crisis Resource Management" (CRM), these CRM skills are represented by intra-team communication, leadership and followership, situational awareness or representation, and teamwork [54]. Each team member must be able to use all available resources, be they information, equipment, or other team members, to achieve the team's common goal of rapid, effective patient care [54].

In this sense, this study's other significant discovery was that students in the simulation group had significantly improved non-technical skills scores compared to the control group regarding all categories of the TEAM scale including teamwork, leadership, and task management (Table 6). Supporting this outcome, Dewolf et al. reported that team-based simulation training improves non-technical skills and can reduce the amount of time needed to perform a simulated cardiac arrest management. Improving non-technical skills can help create more effective teams [55]. Chen et al. noted that teamwork is essential to reducing disruptions during defibrillation and chest compressions, as well as enhancing their efficacy [56]. This emphasizes how crucial teamwork is during times of crisis [57]. Non-technical skills directly affect patient outcomes. There is evidence that non-technical skills (more recently described as power skills) have a direct impact on technical skills. There is a strong relationship between teamwork failure and technical error [58]. Lack of leadership and poor communication lead to critical errors [59], while effective teamwork and communication improve performance and reduce errors [60].

In fact, students practice critical skills including leadership, communication, and teamwork in realistic settings while participating in complex team dynamics during cardiac resuscitation simulations. These simulations also include unforeseen difficulties that instructors provide, encouraging collaboration and adaptability [61].

As a result, given the complexity of the healthcare environment, nurses must become acquainted with the duties and responsibilities of their colleagues in other professions and be proficient in multidisciplinary team communication and practice [62]. Consequently, the nursing work process made a great demand on the development of collaborative skills [63]. Simulation-based interprofessional education can offer a supportive learning environment to enhance collaboration skills and service quality, according to a recent meta-analysis [64].

Additional research has demonstrated that simulation is an effective tool for teaching team dynamics and technical and non-technical skills and, when combined with non-technical skills, enhances technical skill development [65]. When non-technical skills were incorporated into the resuscitation simulation, there were notable improvements in the timeliness of crucial initial steps. Furthermore, effective task management and leadership skills [66] had a positive impact on overall technical performance. In the same way, teamwork during the simulation enhanced group dynamics and output [55,66].

Strengths and limitations

This study results from a strong belief from the authors that learning-based simulation is superior to traditional training for teaching CPR. However, conducting a randomized study is necessary to bring evidence specially to decision-makers in a way that simulation should be integrated systematically within the program. This study is a first at evaluating the impact of simulation as an innovative educational method on the acquisition and retention of knowledge and the development of technical and non-technical skills of nursing students at the HINPHT of Fez, Morocco. The results are important to consider despite the limited sample. This work can be reproduced on a larger scale with a larger sample that would include participants from all institutes in Morocco as part of a multicenter study. Thus, in this study, the retention of CPR knowledge was assessed over a period of 30 days. It would nevertheless be relevant to extend these assessments beyond this duration by also assessing the retention of technical and non-technical skills. In addition, further studies comparing the effectiveness of different simulation and training methods are needed for the development of knowledge and clinical skills of nursing students.

## Conclusions

High-fidelity simulation-based CPR training improved nursing students' acquisition and retention of theoretical knowledge. Additionally, the high-fidelity simulation was determined to have a positive effect on the development of students' technical and non-technical skills. The results of this study support the implementation of simulation as a dynamic teaching strategy. It is suggested that training be repeated periodically in order to progress and update nursing students' knowledge and skills in CPR and nursing in general.
